# A Novel WRKY Transcription Factor from *Ipomoea trifida*, ItfWRKY70, Confers Drought Tolerance in Sweet Potato

**DOI:** 10.3390/ijms23020686

**Published:** 2022-01-08

**Authors:** Sifan Sun, Xu Li, Shaopei Gao, Nan Nie, Huan Zhang, Yufeng Yang, Shaozhen He, Qingchang Liu, Hong Zhai

**Affiliations:** 1 Key Laboratory of Sweet Potato Biology and Biotechnology, Ministry of Agriculture and Rural Affairs/Beijing Key Laboratory of Crop Genetic Improvement/Laboratory of Crop Heterosis & Utilization and Joint Laboratory for International Cooperation in Crop Molecular Breeding, Ministry of Education, College of Agronomy & Biotechnology, China Agricultural University, Beijing 100193, China; sunsifan@cau.edu.cn (S.S.); nongdalixu@126.com (X.L.); spgao@cau.edu.cn (S.G.); chentangtang426@cau.edu.cn (N.N.); zhanghuan1111@cau.edu.cn (H.Z.); yyfyyf5@163.com (Y.Y.); sunnynba@cau.edu.cn (S.H.); liuqc@cau.edu.cn (Q.L.); 2 Cereal Crops Research Institute, Henan Academy of Agricultural Sciences, Postgraduate T&R Base of Zhengzhou University, Zhengzhou 450000, China; 3 School of Agricultural Sciences, Zhengzhou University, Zhengzhou 450000, China

**Keywords:** sweet potato, *Ipomoea trifida*, *ItfWRKY70*, drought tolerance, ABA, stomatal aperture

## Abstract

WRKY transcription factors are one of the important families in plants, and have important roles in plant growth, abiotic stress responses, and defense regulation. In this study, we isolated a WRKY gene, *ItfWRKY70*, from the wild relative of sweet potato *Ipomoea trifida* (H.B.K.) G. Don. This gene was highly expressed in leaf tissue and strongly induced by 20% PEG6000 and 100 μM abscisic acid (ABA). Subcellar localization analyses indicated that ItfWRKY70 was localized in the nucleus. Overexpression of *ItfWRKY70* significantly increased drought tolerance in transgenic sweet potato plants. The content of ABA and proline, and the activity of SOD and POD were significantly increased, whereas the content of malondialdehyde (MDA) and H_2_O_2_ were decreased in transgenic plants under drought stress. Overexpression of *ItfWRKY70* up-regulated the genes involved in ABA biosynthesis, stress-response, ROS-scavenging system, and stomatal aperture in transgenic plants under drought stress. Taken together, these results demonstrated that *ItfWRKY70* plays a positive role in drought tolerance by accumulating the content of ABA, regulating stomatal aperture and activating the ROS scavenging system in sweet potato.

## 1. Introduction

The growth of plants is constantly challenged by abiotic stress conditions such as drought, heat, cold and salinity [1,2]. Drought stress causes severe damage to plants and reduces crop yield, and droughts are likely to be more severe and long lasting because of global warming [3,4]. Therefore, it is urgent to improve the tolerance to drought stress for enhancing crop productivity. Genetic engineering has great potential in improving drought tolerance in plants [5,6,7].

Transcription factors (TFs) are generally the earliest response to abiotic stresses; they act as significant coordinators of the transmission signal and regulate the expression of downstream stress-responsive genes [8]. To date, extensive evidence has shown that many TF families (such as NAC, MYB, bHLH and WRKY) participate in the regulation of stress responses in plants [9,10,11,12]. Among these TF families, the WRKY family is one of the largest families in plants, and plays an important role in plant growth and abiotic stresses [13]. WRKY proteins can be classified into three major groups based on the numbers of WRKY domains and the type of zinc finger motif. Group I proteins contain two WRKY domains and one C_2_H_2_ zinc finger motif. Group II proteins contain one WRKY domain and the same zinc finger motif as group I. This group can be further divided into five subgroups (IIa, IIb, IIc, IId, and IIe). Group III proteins contain one WRKY domain and one C_2_HC zinc finger motif (CX_7_CX_23_HX_1_C) [14]. The function of several WRKY TFs in drought stress has been demonstrated in some kinds of plants. In *Arabidopsis*, *AtWRKY11*, *AtWRKY17*, *AtWRKY28*, *AtWRKY30*, and *AtWRKY63* have been shown to play a positive regulation role in drought tolerance, while *AtWRKY46*, *AtWRKY54* and *AtWRKY70* play a negative regulation role in drought tolerance [15,16,17,18,19]. Overexpression of *TaWRKY1*, *TaWRKY2*, *TaWRKY19*, *TaWRKY33*, *ZmWRKY40, TaWRKY75*, *ZmWRKY106* and *TaWRKY146* enhances tolerance to drought stress in transgenic *Arabidopsis* plants [20,21,22,23,24,25]. Overexpression of *PbrWRKY53* improved tolerance to drought stress in *Pyrus betulaefolia* [26].

The hexaploidy sweet potato (*Ipomoea batatas* (L.) Lam., 2n = 6x = 90) is an important food, a source of bioenergy, and an efficient health-care crop [6,27]. Its productivity is often restricted by drought stress. Because its highly heterozygous, generally self-incompatible, and outcrossing polyploidy, the conventional breeding in sweet potato faces numerous challenges [28]. Genetic engineering is an effective way to improve the drought tolerance of sweet potato [5,29,30,31,32]. However, there are few reports about TFs confering tolerance to drought in sweet potato [30,32,33,34,35]. Diploid *Ipomoea trifida* (H.B.K.) G. Don. (2n = 2x = 30) has been shown to be the most likely diploid ancestor of sweet potato, and is one of the most important sources of resistance genes for sweet potato breeding because of its high resistance to biotic and abiotic stress and low chromosome number [36,37,38]. In this study, a novel WRKY gene, *ItfWRKY70*, was cloned from *I. trifida*, and its overexpression enhanced drought tolerance in transgenic sweet potato.

## 2. Results

### 2.1. Cloning and Sequence Analysis of ItfWRKY70 and Its Promoter

**Figure 1:**
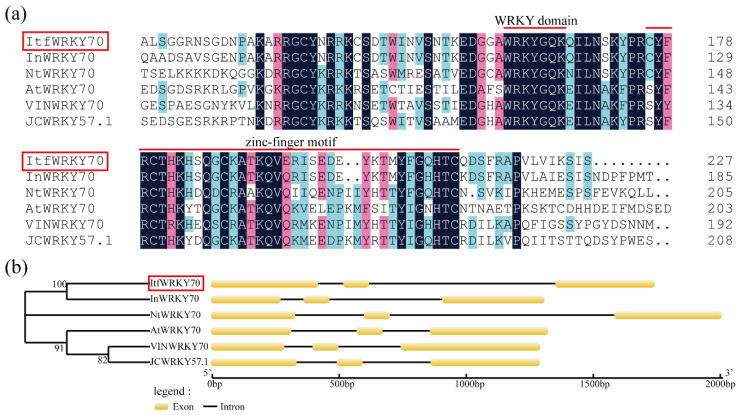
Sequence analysis of ItfWRKY70. (**a**) Multiple sequence alignment of the ItfWRKY70 protein and its closest homologs from different plant species. The WRKY domain and zinc-finger motif are represented with red lines. (**b**) Genomic structures of *ItfWRKY70* and its closest homologs from different plants species. Exons are represented by ellipses, and introns are represented by lines.

The *ItfWRKY70* cDNA sequence was 1063 bp in length and contained a 915 bp open reading frame (ORF) that encoded a predicted polypeptide of 304 amino acids with a calculated molecular weight (MW) of 33.9 kDa and an isoelectric point (pI) of 7.57. This protein had a highly conserved WRKYGQK domain and C_2_HC-type (CX_7_CX_21-23_HX_1_C) zinc finger motif, and it belonged to group III of the WRKY family. It had a high sequence identity with WRKY70 in *Ipomoea nil* (XP_019191673, 60.70%), *Nicotiana tomentosiformis* (XP_009601871, 25.87%), *Vitis vinifera* (XP_002275401, 26.11%) and *Arabidopsis thaliana* (NP_191199.1, 20.06%) (Figure 1a). Phylogenetic analysis showed that ItfWRKY70 has a close relationship with that of *Ipomoea nil* (Figure 1b). The 1748-bp genomic DNA of *ItfWRKY70* contained 3 exons and 2 introns (Figure 1b). A 1668 bp fragment corresponding to the promoter of *ItfWRKY70* was isolated from *I. trifida* genomic DNA and analyzed by using the online analysis software PlantCARE. The result showed that this promoter region contained several kinds of *cis*-acting regulatory elements, which are involved in different abiotic stresses, such as LTR, ARE, GARE and CGTCA-motif (Figure S1).

### 2.2. The Expression of ItfWRKY70 in I. trifida

**Figure 2:**
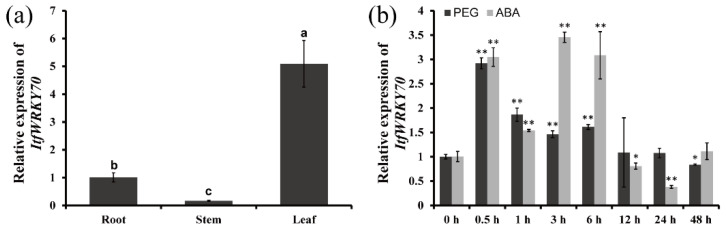
Expression analysis of *ItfWRKY70*. (**a**) Expression analysis of *ItfWRKY70* in root, stem and leaf tissues of *I. trifida*. (**b**) Expression analysis of *ItfWRKY70* in whole plants of *I. trifida* after different times (h) in response to 20% PEG6000 and 100 μM ABA, respectively. Data are presented as means ± SE (*n* = 3). * and ** indicate a significant difference compared to the wild type (WT) at *p* < 0.05 and *p* < 0.01 based on Student’s *t*-test, respectively.

To investigate the potential working site of *ItfWRKY70* in *I. trifida*, we analyzed its expression levels in different tissues, including the root, stem and leaf. The results showed that the *ItfWRKY70* gene exhibited a higher expression level in the leaves of *I. trifida* than that in the stems and roots (Figure 2a). To further analyze its potential function, the expression of *ItfWRKY70* was checked using the whole plants of 4-week-old *I. trifida*, grown in vitro, that were treated with 20% PEG6000 and 100 μM ABA for 0, 0.5, 1, 3, 6, 12, 24 and 48 h. These results showed that the expression of *ItfWRKY70* was significantly induced by 20% PEG and ABA, and peaked at 0.5 h with 2.91-fold, and 3 h with 3.45-fold, respectively (Figure 2b).

### 2.3. ItfWRKY70 Is Localized in the Nucleus

**Figure 3:**
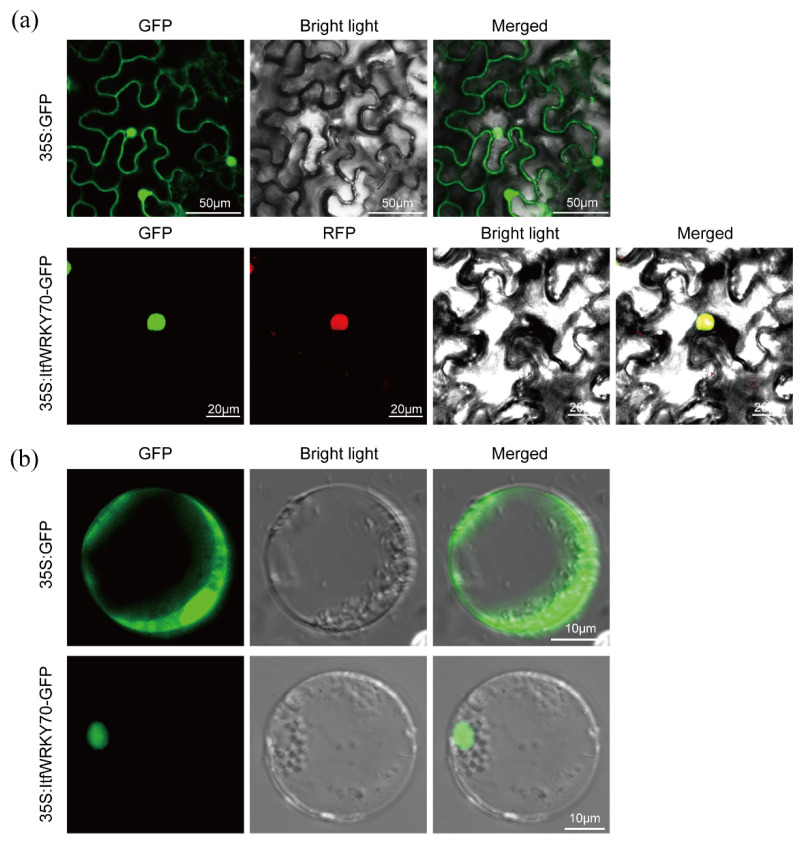
Subcellular localization of ItfWRKY70 in tobacco leaf epidermal cells (**a**) and rice protoplasts (**b**). Confocal scanning microscopic images showed that the ItfWRKY70-GFP fusion protein localized in nuclei vs. GFP as the control.

Subcellular localization of ItfWRKY70 was investigated by examining a ItfWRKY70-GFP fusion protein. The ORF of *ItfWRKY70* controlled by the 35S promoter was fused with GFP, generating a fusion construct ItfWRKY70-GFP. The ItfWRKY70-GFP and red fluorescence protein (RFP)-NLS were transiently expressed in *Nicotiana benthamiana* leaf epidermal cells using *Agrobacterium tumefaciens* (*A. tumefaciens*)-meditated transformation. A nuclear localization signal (NLS) fused to an RFP protein was used in this study as a positive control. Confocal scanning microscopic images from *Nicotiana benthamiana* leaf epidermal cells showed that the green fluorescence emitted by ItfWRKY70-GFP was perfectly overlapped with the red fluorescence of RFP-NLS, indicating that ItfWRKY70 was localized in the nucleus (Figure 3a). We further analyzed the subcellular localization of the ItfWRKY70 in rice protoplasts, and the result was consistent with the above result (Figure 3b).

### 2.4. ItfWRKY70 Has Self-Transcriptional Activation Activity in Yeast

**Figure 4:**
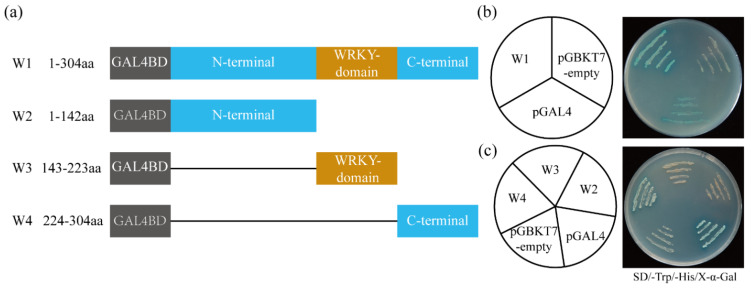
The transcriptional activity of ItfWRKY70 in yeast. (**a**) Schematic diagrams of multiple truncated ItfWRKY70 constructions for transactivation assay in yeast cells. W1, the full length of ItfWRKY70; W2, N-terminal of ItfWRKY70 with 142 amino acid residues; W3, WRKY domain of ItfWRKY70 with 80 amino acid residues; W4, C-terminal of ItfWRKY70 with 80 amino acid residues. (**b**) Transactivation activity assay of full-length in yeast. (**c**) Transactivation activity assay of different ItfWRKY70 mutants in yeast. The pGBKT7 empty vector and pGAL4 were used as negative and positive controls, respectively.

The pGBKT7-ItfWRKY70 fusion construct (W1), the pGBKT7 empty vector (as negative control), and the pGAL4 (as positive control) were separately transformed into the yeast strain AH109 (Figure 4a). Yeast cells containing any of the three vectors grew well on SD/-Trp medium; meanwhile, yeast cells containing W1 and pGAL4 vectors grew well on SD/-Trp/-His/X-α-Gal medium with α-galactosidase activity, whereas containing pGBKT7-empty vector did not grow (Figure 4b). To further analyze which region of ItfWRKY70 protein had the self-activation activity, different truncation construct vectors (W2: a WRKY domain and C-terminal deletion mutant, W3: an N-terminal and C-terminal deletion mutant, and W4: an N-terminal and WRKY domain deletion mutant) were separately transformed into the yeast strain AH109. Yeast cells containing the W4 and positive control grew well on SD/-Trp/-His/X-α-Gal (Figure 4c). These results indicated that deletion of the C-terminal of ItfWRKY70 resulted in loss of its self-transcriptional activation function. This result was consistent with the result of Li et al. [39].

### 2.5. Overexpression of ItfWRKY70 Enhances Drought Tolerance in Sweet Potato

**Figure 5:**
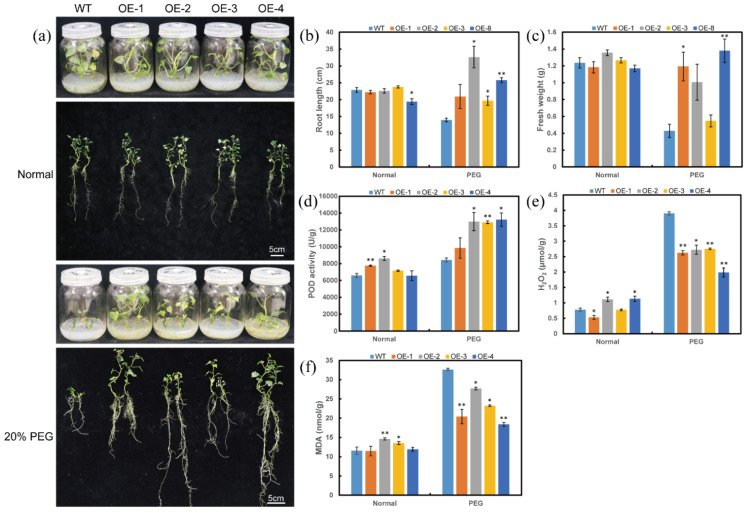
Responses of transgenic and WT sweet potato plants cultured for 4 weeks on MS medium without stress (normal), or 20% PEG6000. (**a**) Phenotypes, (**b**) Root length, (**c**) Fresh weight, (**d**) POD activity in the transgenic and WT plants, (**e**) H_2_O_2_ content, (**f**) MDA content. Data are presented as the means ± SE (*n* = 3). * and ** indicate a significant difference compared to the WT at *p* < 0.05 and *p* < 0.01 based on Student’s *t*-test, respectively.

To further investigate whether *ItfWRKY70* contributes to drought resistance, we generated four overexpression lines (OE-1 to OE-4) of sweet potato. The expression level of *ItfWRKY70* in transgenic plants was significantly higher than that in wild-type (WT) control and there were no significant morphological differences in the aboveground portion and storage root between WT and transgenic plants grown in the field (Figure S2). Transgenic and WT sweet potato plants were cultured on MS medium with 0 or 20% PEG6000 for 4 weeks, respectively. Transgenic and WT sweet potato plants cultured on MS medium without stress showed no differences in growth and rooting. In contrast to the poorly growing WT, the transgenic plants showed vigorous growth and rooting on MS medium with 20% PEG 6000 (Figure 5a–c). POD activity was significantly higher, while H_2_O_2_ and MDA content were significantly lower in the transgenic plants than in WT (Figure 5d–f).

**Figure 6:**
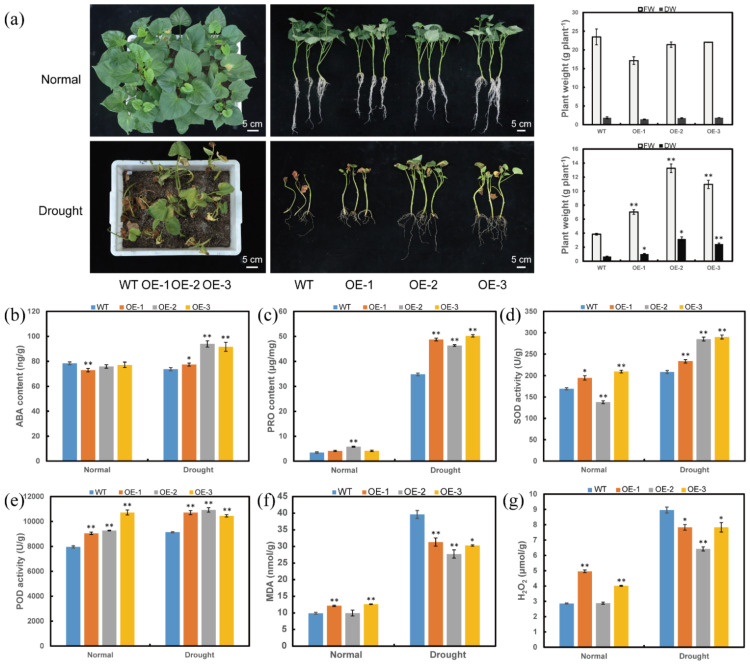
Responses of transgenic and WT sweet potato plants grown in a transplanting box with no stress (normal) and drought stress. (**a**) Phenotypes, FW and DW, (**b**) ABA content, (**c**) Proline content, (**d**) SOD activity, (**e**) POD activity, (**f**) MDA content, (**g**) H_2_O_2_ content. The phenotypes are shown after drought treatment for 6 weeks. FW, fresh weight; DW, dry weight. Data are presented as the means ± SE (*n* = 3). * and ** indicate a significant difference compared to the WT at *p* < 0.05 and *p* < 0.01 based on Student’s *t*-test, respectively.

Furthermore, the transgenic plants and WT were grown in a transplanting box, and then treated with drought. There were no differences in growing and rooting among the transgenic and WT plants under normal conditions. Under drought stress, the *ItfWRKY70*-OE lines exhibited better growth and lager FW and DW than the WT plants (Figure 6a). The transgenic lines showed increased content of ABA and proline, increased activities of SOD and POD, and decreased content of MDA and H_2_O_2_ than that in WT plants under drought stress (Figure 6b–g). These results indicated that overexpression of *ItfWRKY70* enhances drought tolerance in sweet potato.

### 2.6. Stomatal Movement in Leaves

**Figure 7:**
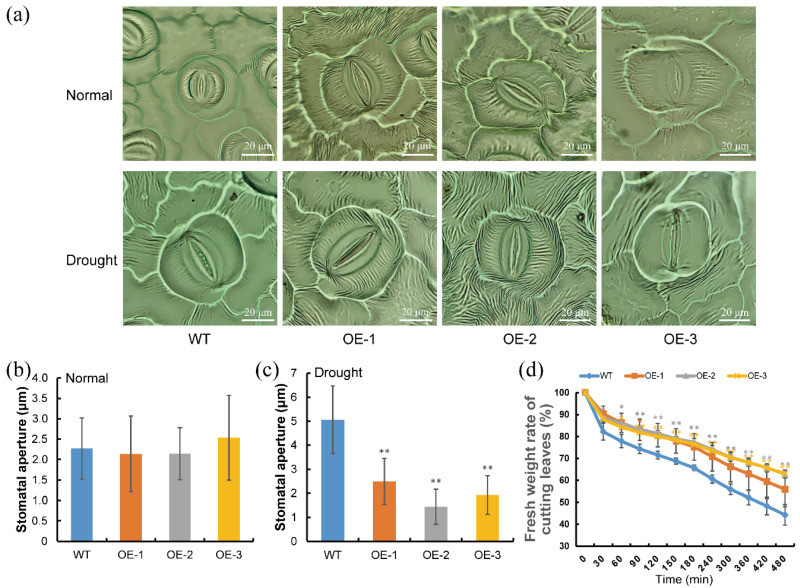
Stomatal aperture and water loss in transgenic lines and WT plants. (**a**) Observation of stomata in different plant leaves under normal and drought stress. (**b**,**c**) Stomatal apertures were measured using ImageJ software. (**b**) Normal condition and (**c**) Drought condition. Data are presented as the means ± SE (*n* > 40). (**d**) The fresh weight rate of cutting leaves in OE-1, -2, -3, and WT under dehydration treatment. Error bars represent sd (*n* = 3). *p*-values of significant difference of transgenic lines compared to the WT based on Student’s *t*-test, respectively (Supplementary Table S1). * and ** indicate a significant difference compared to the WT at *p* < 0.05 and *p* < 0.01 based on Student’s *t*-test, respectively.

Transgenic and WT plants were treated with drought stress without water for 4 weeks. Then the leaves of these plants were analyzed for stomatal observation. Stomatal apertures of transgenic plants showed no significant difference with WT under no stress, whereas stomatal apertures of transgenic lines, OE-1, OE-2 and OE-3, were smaller, by about 50%, 72% and 62%, than those of WT under drought stress, respectively (Figure 7a–c). Further analysis results showed that the leaves of transgenic plants experienced a higher water content than that in WT plants with the increase of dehydration treatment time (Figure 7d). These results suggested that *ItfWRKY70* might play an important role in reducing water evaporation by controlling stomatal aperture.

### 2.7. Overexpression of ItfWRKY70 Upregulates the Expression of the Stress-Responsive Genes

**Figure 8:**
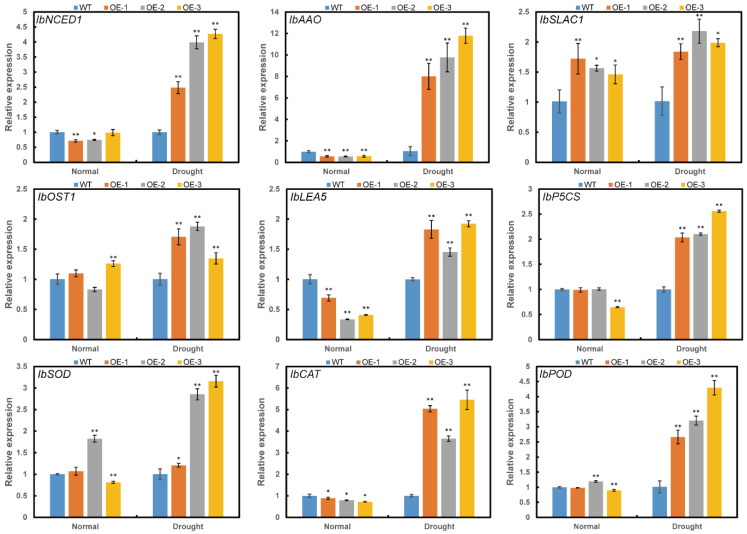
Expression levels of stress-responsive genes in transgenic and WT sweet potato plants. Plants grown in the transplanting boxes were sampled for analysis after treating with no stress (normal) for 4 weeks, drought stress for 4 weeks. Data are presented as the means ± SE (*n* = 3). * and ** indicate a significant difference compared to the WT at *p* < 0.05 and *p* < 0.01 based on Student’s *t*-test, respectively.

To investigate the reason that *ItfWRKY70* affected drought resistance in transgenic plants, we analyzed the expression of several genes involved in different pathways. Genes involved in ABA biosynthesis: 9-cis-epoxycarotenoid dioxygenase (*NCED*) and aldehyde oxidase (*AAO*), proline biosynthesis (*P5CS*), late embryogenesis abundant protein (*LEA*), ROS-scavenging system (*SOD*, *POD*, and *CAT*), stomatal movement: SLOW ANION CHANNEL-ASSOCIATED 1 (*SLAC1*) and *OST1/SnRK2.6* were significantly up-regulated in the transgenic sweet potato plants compared with WT under drought stress (Figure 8).

## 3. Discussion

### 3.1. Overexpression of ItfWRKY70 Enhances Drought Tolerance

WRKY TFs act as one of the important families in plants, and have been shown to be necessary for plant growth, signal transduction, and abiotic stress responses [13,14]. However, so far, there are few reports about *WRKY70* involved in drought stress in plants. The overexpression of *FcWRKY70* enhanced tolerance to drought in tobacco [40]. *MfWRKY70*-overexpression in *Arabidopsis* plants increased drought tolerance [41]. The *wrky46wrky54wrky70* mutant enhanced the drought tolerance in *Arabidopsis* [19]. Moreover, few studies have focused on the role of *WRKY* genes in sweet potato and *I. trifida*, with the exception of *IbWRKY2* and *ItWRKY1* [37,42].

Sweet potato (*I. batatas*) is an important food crop; however, its improvement by conventional breeding is limited because its highly heterozygous, generally self-incompatible and outcrossing polyploidy [43]. Genetic engineering is used to improve the sweet potato [5,29,30,31,32]. The wild ancestor diploid *I. trifida* acts as an effective resource to improve sweet potato because of its high resistance to abiotic stress [37]. In this study, we cloned a novel *WRKY* gene, *ItfWRKY70*, from the wild progenitor of sweet potato, *I. trifida*. The *ItfWRKY70* showed a higher expression level in leaves, and was significantly induced under 20% PEG and ABA treatment (Figure 2b). Its overexpression significantly enhanced the tolerance to drought in transgenic sweet potato (Figure 5 and Figure 6).

### 3.2. Overexpression of ItfWRKY70 Upregulates Stress-Responsive Genes

TFs usually act as regulators and molecular switches in regulating the expression levels of stress-responsive genes [9,14]. Thus, TFs often have transcriptional activation activity or transcriptional repression activity. Different WRKY TFs have different transcriptional activity domain (N-terminal or C-terminal, but not WRKY domain). The N-terminal region of IbWRKY2 is the self-transcriptional activation domain detected in yeast cells [42]. The C-terminal domain of TaWRKY46, the homologous protein of WRKY70, has an important role in self-transcriptional activation function detected in yeast [39]. In our study, we also found that the C-terminal region of ItfWRKY70 functioned as a transcriptional activator (Figure 4).

**Figure 9:**
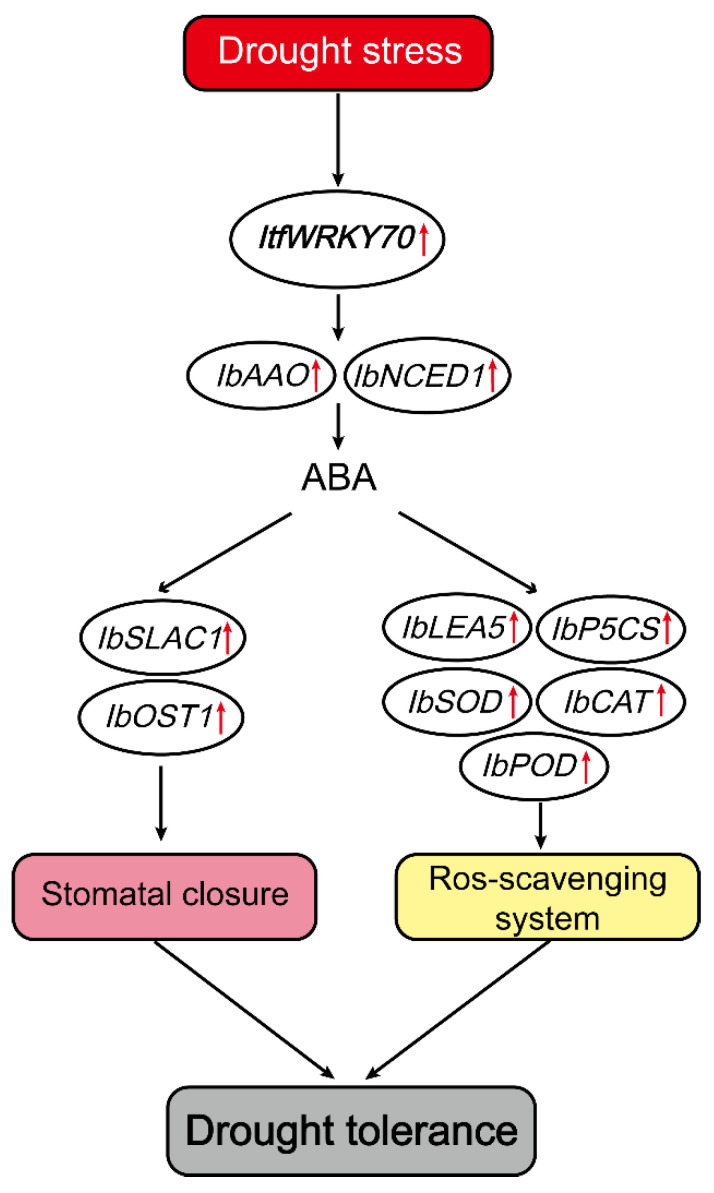
A proposed model for regulation of ItfWRKY70 in drought stress tolerance in transgenic sweet potato. **↑** Indicates up-regulation of genes coding these enzymes (proteins).

ABA acts as chemical signal and plays an important role in regulating the adaptive response of plants to abiotic stresses [44,45]. It has been reported that *NCED1* and *AAO* are responsible for ABA accumulation [46]. Overexpression of *CrNCED1* in transgenic tobacco displayed enhanced tolerance to drought stress via increasing ABA content [47]. ABA has been reported to regulate the expression levels of stress-tolerance-related genes, including *P5CS*, *LEA*, *SOD*, and *POD*, in several plant species [31,48,49,50]. Overexpression of *FcWRKY70* conferred drought tolerance in tobacco (*Nicotiana nudicaulis*) and lemon (*Citrus lemon*) by reducing water loss and regulating the expression level of *ADC* [40]. The overexpression of *MfWRKY70*, a homology gene of *ItfWRKY70*, upregulated stress-associated genes (*P5CS, NCED3* and *RD29A*), and maintained ROS homeostasis, leading to increased drought tolerance in transgenic *Arabidopsis* plants [41]. In this study, the expression of *NCED1* and *AAO* involved in ABA synthesis and the content of ABA significantly increased in transgenic plants compared with WT under drought stress. The expression levels of *P5CS* and *LEA* was significantly up-regulated in transgenic plants (Figure 8). These results indicated that *ItfWRKY70* might play an important role in the regulation of the stress-responsive gene via the ABA signaling pathway (Figure 9).

### 3.3. Overexpression of ItfWRKY70 Enhances the ROS-Scavenging System

It is well known that abiotic stresses lead to the excessive production of reactive oxygen species (ROS) [51]. The high H_2_O_2_ and O_2_^−^ content could seriously damage the plants, and affect the development and productivity of the crop [52,53]. To escape the damage from ROS, plants have evolved a complex ROS-scavenging system to protect plants from ROS [54]. *MfLEA* overexpression in tobacco enhanced tolerance to drought, cold, and high-light stress by reducing the accumulation of ROS [55]. Overexpression of *Mn-SOD* improved salt tolerance by inhibiting ROS accumulation in *Arabidopsis thaliana* plants [56]. Overexpression of *GsPOD40* in soybean enhanced tolerance to drought stress through alleviation of ROS induced oxidative damage [57]. In this study, the expression levels of the ROS-scavenging system genes and the activity of SOD and POD were significantly increased, and the content of H_2_O_2_ decreased in transgenic plants compared with WT under drought stress (Figure 6 and Figure 8). These results suggest that *ItfWRKY70* confers drought tolerance by activating the ROS-scavenging system in sweet potato.

### 3.4. Overexpression of ItfWRKY70 Regulates Stomatal Movement

The stoma of leaves play an important role in the water loss of plants, and water vapor loss in mature leaves depends on the stomatal size and density [58]. Plants with less water loss usually have greater drought tolerance under drought conditions [59]. ABA also plays a critical role in the regulation of plant stomatal behavior [60,61]. Extensive evidence indicates that water loss in plants is explicitly linked with stomatal movement and drought tolerance. The overexpression of *OsWRKY45* in plants caused a higher content of water and higher survival rate than WT by closing stomata under drought conditions [62]. Similarly, overexpression of *TaWRKY146* enhances drought tolerance by reducing stomatal closure in *Arabidopsis thaliana* [23]. A previous study reported that *SLAC1* is necessary for ABA-mediated stomatal closure [63]. In *ost1* mutations, the deletion of *OST1* gene disrupted ABA induction of stomatal closure under drought stress [61]. In this study, we found that *ItfWRKY70* was more highly expressed in leaf tissues than in root and stem tissues (Figure 2a). Under dehydration conditions, the stoma of transgenic plants leaves exhibited smaller stomatal aperture sizes and the leaves of transgenic plants experienced a higher water content than in WT plants (Figure 7a–c). The genes related to stomatal movement, *IbSLAC1* and *IbOST1/SnRK2.6*, had high expression levels in transgenic sweet potato plants (Figure 8). These results indicated that *ItfWRKY70* improves drought tolerance by affecting stomatal movement (Figure 9).

## 4. Materials and Methods

### 4.1. Plant Materials and Growth Conditions

The wild relative of sweet potato, *I. trifida*, was used to isolate the *ItfWRKY70* gene. The sweet potato cultivar Lizixiang was used for characterizing the function of *ItfWRKY70*. The plants were cultured on Murashige and Skoog (MS) medium for 4 weeks at 27 ± 1 °C under 13 h of daylight at 54 μmol m^−2^s^−1^.

### 4.2. Cloning and Sequence Analysis of ItfWRKY70

Total RNA of *I.trifida* was extracted using the RNAprep Pure Plant Kit (Tiangen Biotech, Beijing, China), and the first-strand cDNA synthesis was performed using PrimeScript^TM^ II 1st Strand cDNA Synthesis Kit (TaKaRa, Beijing, China). According to the EST obtained in a previous study [64] and referring to the genomic data of *I. trifida* (http://sweetpotato.uga.edu/, accessed on 12 February 2020), the cDNA sequence of *ItfWRKY70* gene was obtained with a primer pair (*ItfWRKY70*-ORF-F/R) and homology-based cloning method. All of the special primers are listed in Supplementary Table S2. The MW and pI of *ItfWRKY70* were calculated with ExPASy (https://web.expasy.org/compute_pi/, accessed on 24 February 2020). *ItfWRKY70* was analyzed with online BLAST (https://blast.ncbi.nlm.nih.gov/Blast.cgi, accessed on 15 March 2020). Multiple protein sequences of ItfWRKY70 were determined with the DNAMAN software (LynnonBiosoft, San Ramon, CA, USA). A phylogenic tree was constructed using MEGA 5.0 software (https://www.megasoftware.net/, accessed on 15 March 2021) with the neighbor-joining method. The exon-intron structure was constructed using GSDS2.0 (http://gsds.gao-lab.org/, accessed on 16 March 2020). The *cis*-acting regulatory elements in the promoter region of *ItfWRKY70* were screened with PlantCARE (http://bioinformatics.psb.ugent.be/webtools/plantcare/html/, accessed on 5 April 2020).

### 4.3. Expression Analysis of ItfWRKY70

The transcript levels of ItfWRKY70 in the leaves, stems and roots were measured with untreated *I. trifida* plants. The 4-week-old in vitro grown-plants were treated in Hoagland solution with 20% PEG6000 and 100 μMABA, respectively. The whole plants were sampled at 0 h, 0.5 h, 1 h, 3 h, 6 h, 12 h, 24 h, and 48 h after treatment. The expression of *ItfWRKY70* was measured using primer pairs (qRT-*ItfWRKY70*-F/R) of *ItfWRKY70* and *ItfGAPDH* (itf07g03920.t1) as internal control (Supplementary Table S2) as described by Li et al. [65].

### 4.4. Subcellular Localization

The encoding regions of ItfWRKY70 without stop codon was amplified using a primer pair (1300-*ItfWRKY70*-GFP-F/R) and integrated into the expression vector pCAMBIA1300-GFP (Supplementary Table S2). The recombinant vectors p35S:ItfWRKY70-GFP, p35S:GFP (as control), and p35S:RFP-NLS (Nuclear maker) were transformed into *A. tumefaciens* strain EHA105 by heat shock method, respectively, and transiently expressed in *Nicotiana benthamiana* leaf epidermal cells using Agrobacterium infiltration [66]. The GFP and RFP inflorescence signals were observed with a confocal laser-scanning microscope LSM880 (Zeiss, Oberkochen, Germany) after 48 h of growth.

Isolation of rice protoplasts and transfection of the vectors into the protoplasts were performed according to the method of Yoo et al. [67]. The p35S:ItfWRKY70-GFP and p35S:GFP constructs were transfected into the isolated protoplasts, respectively. The GFP inflorescence signals were observed by LSM880 (Zeiss, Oberkochen, Germany) after 16~18 h incubation.

### 4.5. Transcriptional Activation Assay

The full-length and various deletion fragments (a WRKY domain and C-terminal deletion mutant, an N-terminal and C-terminal deletion mutant, and an N-terminal and WRKY domain deletion mutant) of *ItfWRKY70* were amplified by PCR using the primer pairs pGBKT7-ItfWRKY70-F/R, pGBKT7-ItfWRKY70-F/-1-R, pGBKT7-ItfWRKY70-2-F/R, and pGBKT7-ItfWRKY70-3-F/pGBKT7-ItfWRKY70-R (Supplementary Table S2) and ligated to the *Nde*I/*EcoR*I-digested pGBKT7 vector to produce the fusion construct vectors (pGBKT7-W1, -W2, -W3, and -W4), respectively. The empty pGBKT7 vector was used as negative control, and pGAL4 was used as a positive control. The fusion plasmid, positive control and negative control were transformed into the yeast strain AH109, respectively. The transformed yeast was streaked on SD/-Trp and SD/-Trp/-His/X-α-Gal plates to observe yeast growth at 30 °C for 2–3 days.

### 4.6. Production of Transgenic Sweet Potato Plants

The coding region of *ItfWRKY70* was inserted into pCAMBIA1300. The recombinant vector was transferred into *A. tumefaciens* strain EHA105. Transformation and plant regeneration were performed by the *A.tumefaciens*-mediated transformation as previously described by Liu et al. [68] and Zhai et al. [31]. The putative transgenic sweet potato plants were identified by PCR analysis with qRT-PCR primers (Supplementary Table S2). The transgenic and the WT plants were transferred to soils in a greenhouse and then in a field for their evaluation. The cuttings about 25 cm in length were used for further function analysis as described by Zhai et al. [31] and Zhang et al. [32].

### 4.7. Assay for Drought Tolerance

In vitro-grown ItfWRKY70-overexpression and WT sweet potato plants were cultured on MS medium with 20% PEG 6000. After 4 weeks, the growth was continuously observed for 4 weeks. Proline and MDA contents and superoxide dismutase (POD) activity were analyzed using Assay Kits (Comin Biotechnology Co., Ltd. Suzhou, China).

For further tolerance evaluation, the 25-cm-long cuttings from transgenic and WT plants grown in the field for 6 weeks were planted in a transplanting box in a greenhouse. These plants were irrigated with half-Hoagland solution for one week. Then they were treated with drought stress without water for 6 weeks. Three cuttings were treated for each line. FW and DW were measured after 6 weeks. Meanwhile, proline, H_2_O_2_ and MDA contents, SOD and POD activity in the leaves of transgenic and WT plants were analyzed using Assay Kits (Comin Biotechnology Co., Ltd. Suzhou, China) and ABA contents was measured with indirect enzyme-linked immunosorbent assay (ELISA) [69].

### 4.8. Observation of Leaf Stomata and Leaf Water Loss Bioassays

Transgenic plants and WT plants were treated with drought stress without water for 6 weeks. Then the fully unfolded leaves at the same position of these plants were selected for stomatal observation. The leaves of abaxial epidermal stripes were peeled away, and the stomata imaged by Echo Revolve light microscopy (ECHO, San Diego, CA, USA). The images were used to estimate stomatal apertures with the help of ImageJ software (downloaded from https://imagej.nih.gov/ij/download.html, accessed on 6 September 2021) according to the method of Lin et al. [70]. About 40 stomatal pores from the same region of leaf were examined for each measurement assay.

For water loss treatment, the fully unfolded leaves at the same position were detached from transgenic and WT plants grown in field for 6 weeks (three replicates per treatment) and put in a 37 °C dry incubator. The leaves were weighed at 0 min, 30 min, 60 min, 90 min, 120 min, 150 min, 180 min, 240 min, 300 min, 360 min, 420 min and 480 min. Kinetic analysis of water loss was performed and represented as the percentage of initial fresh weight at each designated time point [71].

### 4.9. Expression of Stress-Responsive Genes

The cuttings of transgenic lines (OE-1, OE-2, OE-3) and WT plants grown in the transplanting box were treated with no stress (normal) for 4 weeks as control, and drought stress for 4 weeks. Three cuttings were treated for each line. Their leaves were further used to confirm the expression of the genes involved in ABA biosynthesis (*NCED*, *AAO3*), active oxygen scavenging (*SOD*, *CAT* and *POD*), osmotic adjustment substances (*P5CS*, *LEA5*), and stomatal movement (*SLAC1* and *OST1/SnRK2.6*) with gene-specific primers (Supplementary Table S2).

### 4.10. Statistical Analysis

All the experiments were completed with three biological replicates. All data are presented as mean ± SE. Means were compared by student’s *t*-test (two-tailed analysis) at *p* < 0.05 (*) and *p* < 0.01 (**).

## 5. Conclusions

A novel WRKY gene, *ItfWRKY70*, was isolated from the wild relative of sweet potato *I. trifida*. This is the first report that *ItfWRKY70* confers tolerance to drought in sweet potato. Its overexpression enhanced drought tolerance by regulating stress-responsive related genes, regulating stomatal aperture and activating the ROS scavenging system in transgenic sweet potato plants. *ItfWRKY70* might have potential application prospects in improving the drought tolerance of sweet potato and other plants.

## Data Availability

The data presented in this study are available on request from the corresponding author.

## Supplementary Materials

The following supporting information can be downloaded at www.mdpi.com/article/10.3390/ijms23020686/s1.
