# Supplementary material for: A Novel WRKY Transcription Factor from Ipomoea trifida, ItfWRKY70, Confers Drought Tolerance in Sweet Potato
Source: Int J Mol Sci. 2022 Jan 8;23(2):686. doi: 10.3390/ijms23020686 (PMC8775875; doi:10.3390/ijms23020686)
Supplement: Supplementary file 1 [file ijms-23-00686-s001.zip › ijms-1534835-supplementary.pdf]

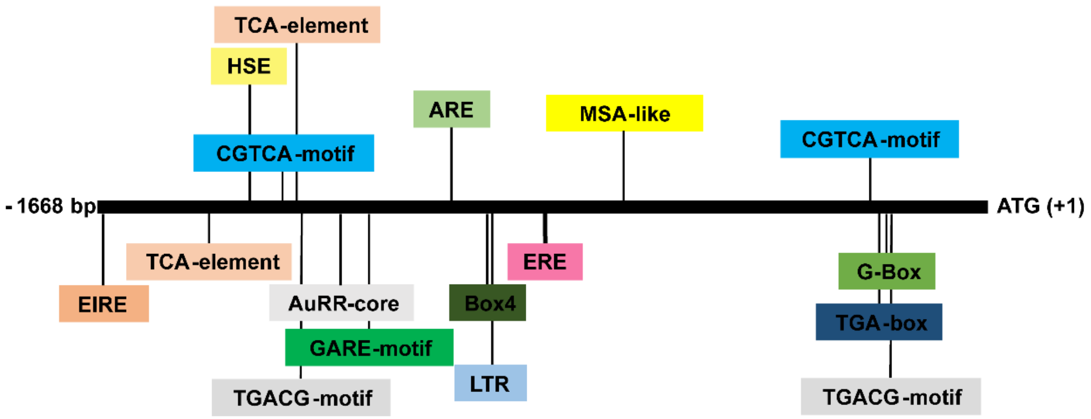

Figure S1. The type and locations of *cis*-acting elements present in the *ItfWRKY70* promoter region. The promoter region, which is 1688 bp upstream of the initiation codon, was used for the analysis. Differently colored boxes represent different cis-acting elements. ATG indicates the start codon of *ItfWRKY70*.

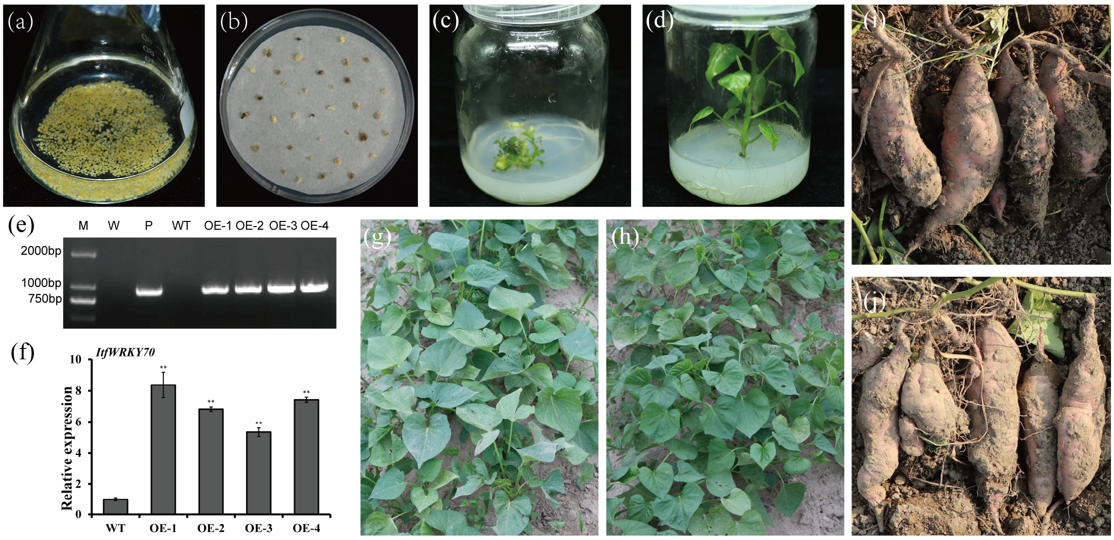

Figure S2. Production of the *ItfWRKY70*-overexpression sweet potato plants. (**a**) Embryogenic suspension cultures rapidly proliferating in MS medium with 2.0 mg l-1 2,4-D. (**b**) Hygromycin (Hyg)-resistant embryogenic calluses formed on MS medium with 2.0 mg l^{-1} 2,4-D, 100 mg l^{-1} Carb and 11 mg l^{-1} Hyg after 8 weeks of selection. (**c**) Regeneration of plants from Hyg-resistant calluses on MS medium with 1.0 mg l^{-1} ABA and 100 mg l^{-1} Carb. (**d**) Transgenic plant cultures in MS medium. (**e**) PCR analysis of transgenic plants. M, DL2000 DNA maker; W, water as a negative control; P, plasmid pCAMBIA1300-*ItfWRKY70* as positive control; WT, wild plant as negative control; OE-1, 2, 3 and 4, transgenic plants. (**f**) Transcript levels of *ItfWRKY70* in the transgenic and WT plants. The results are expressed as relative values with respect to the transcript level of the WT, which was set to 1.0. Data are presented as the means ± SE (n=3). ** indicates a significant difference compared with the WT at $p < 0.01$ based on Student's *t*-test. (**g**, **i**) and (**h**, **j**) WT and transgenic plants grown in a field, respectively.

Supplementary Table S1. P-values of significant difference of transgenic lines compared to the WT based on Student's *t*-test

times	WT vs. OE-1(p-value)	WT vs. OE-2(p-value)	WT vs. OE-3(p-value)
0	1	1	1
30	0.0555667845707	0.047311738691481	0.070802344154136
60	0.054068976618353	0.01152606117809	0.02207567962716
90	0.046280327796457	0.003024896965646	0.004068188780264
120	0.049591192626879	0.001807305001049	0.001473879105401
150	0.050677587918364	0.000546083655797	0.000355955175312
180	0.053577827917223	0.000690411651819	0.000212874826846
240	0.061023040364947	0.000896402743211	0.00045508042211
300	0.070562346053746	0.001125343771288	0.000749161580278
360	0.078647328671946	0.001707608555704	0.001422477775335
420	0.090051581717234	0.00237698949973	0.002008771312636
480	0.093968862340237	0.00309046314194	0.002714831554006

Supplementary Table S2. Primers used in this study

Primer name	Primer sequence (5′-3′)
primers for ORF/genome DNA	
ItfWRKY70-ORF-F	ATGTATGATATTTCTACTGCTTCAA
ItfWRKY70-ORF-R	TTAATAATCCAAGAGTCGCATTTC
primers for promoter of ItfWRKY70	
ItfWRKY70-promoter-F	AATCGGGGTCGAATCTATGAC
ItfWRKY70-promoter-R	TTGTGGAAATTTAAATAAATAAACT
Primers for overexpression vector/subcellular localization	
1300-*ItfWRKY70*-GFP-F	ACGGGGGACGAGCTCGGTACCATGTATGATATTTCTACTGCTTCAA
1300-*ItfWRKY70*-GFP-R	GCTCACCATGTCGACTCTAGAATAATCCAAGAGTCGCATTTC
Primers for identifying overexpression plants	
35s-F	TCCTTCGCAAGACCCTTCCTC
JD-*ItfWRKY70*-R	ATTTACAACCACGTCGTCATAG
Primers for qRT-PCR	
qRT-*ItfWRKY70*-F	AGGGCAAGAAATCAGCCACT
qRT-*ItfWRKY70*-R	AGCCTCGGAGAAAGATCCCA
qRT-*P5CS*-F	GCCTGATGCACTTGTTCAGA
qRT-*P5CS*-R	TTGAGCAATTCAGGGACCTC
qRT-*POD*-F	TTCACGACTGCTTCGTTGA
qRT-*POD*-R	TTCTCAACCGCGGTCTTAA
qRT-*SOD*-F	TCCTGGACCTCATGGATTTC
qRT-*SOD*-R	GCCACTATGTTTCCCAGGTC
qRT-*CAT*-F	ACGCAATTCCCGGACGTGAT
qRT-*CA*T-R	AAGCCTTCCATGTGGCGGTA
qRT-*NCED1*-F	GATTGGTTGGAGTGGGCGAT

qRT-*NCED1*-R	CTTCAGGAGCTGGAGCGAAA
qRT-*AAO*-F	GTCGTTTATGCGGGCTCCT
qRT-*AAO*-R	CCTTTTCGTCCACCGATTTT
qRT-*LEA5*-F	TCCCGTGACCGGATACTACA
qRT-*LEA5*-R	TCTTCAAGAGCATCTGACGCA
qRT-*IbSLAC1*-F	ATACACGACCGCCATCTTCTCC
qRT-*IbSLAC1*-R	TGTCATCATTATCCGCCATTATTT
qRT-*OST1/SnRK2.6*-F	GCTCGTCAAGGACATCGGTTC
qRT-*OST1/SnRK2.6*-R	CTGACAATGTTGGGATGCCTTA
qRT-*Actin*-F	AGCAGCATGAAGATTAAGGTTGTAGCAC
qRT-*Actin*-R	TGGAAAATTAGAAGCACTTCCTGTGAAC
qRT-*SnRK1*-F	TCTTAGTCCCAAGAGAAGAAAAAT
qRT-*SnRK1*-R	TAAATAAAATCTATTCAAGGCAATG
qRT-*ItfNADPH*-F	AGACTCTTCTGTTTGGCGAGAAGT
qRT-*ItfNADPH*-R	AGGAGCAGAGATCACAACCTTCTT
Primers for transactivation activity	
pGBKT7-ItfWRKY70-F	ATCTCAGAGGAGGACCTGCATATGATGTATGATATTTC TACTGCTTCAA
pGBKT7-ItfWRKY70-R	GTCGACGGATCCCCGGGAATTCTTAATAATCCAAGAG TCGCATTTC
pGBKT7-ItfWRKY70-1-R	GTCGACGGATCCCCGGGAATTCCTTTCTTCTGTTGTAA CATCCTCG
pGBKT7-ItfWRKY70-2-F	ATCTCAGAGGAGGACCTGCATATGTGTTCAGATACATG GATCAATGTCT
pGBKT7-ItfWRKY70-2-R	GTCGACGGATCCCCGGGAATTCTTTGATCACGAGAAC TGGAGC
pGBKT7-ItfWRKY70-3-F	ATCTCAGAGGAGGACCTGCATATGTCTATCTCGACTGT GGATTCTACTC
